# Integration of genome and transcriptome reveal molecular regulation mechanism of early flowering trait in *Prunus* genus (*Prunus mume* and *Prunus persica*)

**DOI:** 10.3389/fpls.2022.1036221

**Published:** 2022-10-06

**Authors:** Ping Li, Qin Zhang, Baosheng Shi, Liu Liu, Xiaoman Zhang, Jia Wang, Haihui Yi

**Affiliations:** ^1^ College of Landscape and Tourism, Hebei Agricultural University, Baoding, China; ^2^ National Engineering Research Center for Floriculture, School of Landscape Architecture, Beijing Forestry University, Beijing, China; ^3^ College of Agronomy, Inner Mongolia Minzu University, Tongliao, China

**Keywords:** *Prunus* genus, early flowering, gene mining, multi-omics, molecular model

## Abstract

Flowering time is crucial for the survival and reproduction. *Prunus* genus belongs to the Rosaceae family and includes several hundred species of flowering trees and shrubs with important ornamental and economic values. However, the molecular mechanism underlying early flowering in *Prunus* genus is unclear. Here, we utilized the genome and transcriptome of *P. mume* and *P. persica* to explore the transcriptional regulation mechanism of early flowering. Comparative genomics found that genes accounting for 92.4% of the total *P. mume* genome and 91.2% of the total *P. persica* genome belonged to orthogroups. A total of 19,169 orthogroups were found between *P. mume* and *P. persica*, including 20,431 corresponding orthologues and 20,080 collinearity gene pairs. A total of 305 differentially expressed genes (DEGs) associated with early flowering were found, among which *FT*, *TLI65*, and *NAP57* were identified as hub genes in the early flowering regulation pathway. Moreover, we identified twenty-five transcription factors (TFs) from nine protein families, including MADS-box, AP2/ERF, and MYB. Our results provide insights into the underlying molecular model of flowering time regulation in *Prunus* genus and highlight the utility of multi-omics in deciphering the properties of the inter-genus plants.

## Introduction

Flowering time is regarded as an environmental adaptive trait of plants, which is crucial for the survival and production of offspring (Ding et al., 2020; Gaudinier and Blackman, 2020). The flowering process of plants is influenced by a number of complex external environmental conditions (nutrient conditions, ambient temperature and photoperiod, etc.) and genetic factors (Andrés and Coupland, 2012; Freytes et al., 2021). The early flowering may result in damaged floral organs, insufficient ovule fertilization and less flower production. Late flowering may be detrimental to seed ripening and dispersal (Gaudinier and Blackman, 2020). Therefore, the flowering time directly affects the yield of cash crops and the timing of ornamental plants (Gaudinier and Blackman, 2020).

Plants have evolved complex molecular mechanisms to regulate flowering time (Gaudinier and Blackman, 2020). Autonomous, photoperiod, vernalization, and gibberellin pathways have been widely studied to be involved in flowering regulation in *Arabidopsis thaliana* (Johansson and Staiger, 2015; Xu and Chong, 2018; Sharma et al., 2020; Wu et al., 2020; Quiroz et al., 2021). In addition, age, trehalose 6-phosphate synthase (TPS), and thermosensory pathways have been proposed to be involved in the flowering process (Blázquez et al., 2003; Halliday et al., 2003; Balasubramanian et al., 2006; Wu and Poethig, 2006; Schwarz et al., 2008; Wahl et al., 2013). These pathways have also been studied in other plants, such as *Oryza sativa*, *Glycine max* and *Medicago truncatula* (Weller and Macknight, 2018; Shim and Jang, 2020; Lin et al., 2021). Among the other regulators, these pathways also share some key integrators of flowering time, such as the FLOWERING LOCUS T (FT), SUPPRESSOR OF OVER EXPRESSION OF CONSTANS (SOC1), LEAFY (LFY), and FLOWERING LOCUS C (FLC) (Simpson and Dean, 2002; Posé et al., 2012; Ó'Maoiléidigh et al., 2014; Maurya et al., 2020). The flowering process of woody plants is very different from that of herbaceous plants. Most woody plants have flower buds formed before winter but need a cold duration to flower in the temperate and cold zone (Fuente et al., 2015; Goeckeritz and Hollender, 2021). Therefore, flowering regulation in most woody plants is related to flower bud dormancy and chilling requirement (Luedeling, 2012; Yu et al., 2020). Some genes that regulate bud dormancy and flowering have been identified in woody plants, such as *FT*, *DORMANCY ASSOCIATED MADS-box* (*DAM 1-6*), *SHORT VEGETATIVE PHASE* (*SVP*), and *APETALA1* (*AP1*) (Bielenberg et al., 2008; Jiménez et al., 2009; Canton et al., 2021; Li et al., 2021; Zhang et al., 2021b). However, the complete digging of the molecular mechanisms of flowering in deciduous trees still require more holistic studies.

Recently, the transcriptomic analysis to detect gene expression during flower bud dynamic changes has been widely used in woody plants (Zhang et al., 2018; Rothkegel et al., 2020; Zhang et al., 2021c). *Prunus* genus includes the best representative species, such as *P. persica*, *P. mume*, *Prunus avium*, and *Prunus armeniaca* (Zhang et al., 2018; Rothkegel et al., 2020; Yu et al., 2020; Canton et al., 2021; Zhang et al., 2021c; Canton et al., 2022). So far, 191 candidate genes associated with flowering time traits have been identified in *P. mume* using integrated phenotypic data, genome-wide association study (GWAS), differentially expressed genes (DEGs), and gene co-expression studies (Zhang et al., 2021c). DAM gene family members are significantly associated with flowering time and bud dormancy (Kai et al., 2018; Zhang et al., 2018). DAM gene family members have also been discovered during flower bud development in *P. persica* and *P. armeniaca* (Bielenberg et al., 2008; Leida et al., 2012; Yu et al., 2020). In addition to *DAMs*, other transcription factors and regulatory genes have been found in *Prunus* plants, such as *SVP*, *AP1*, *AGAMOUS-LIKE 24* (*AGL24*), *APETALA3* (*AP3*), *SEPALLATA 1, 2, 3* (*SEP1, 2, 3*), and *PISTILLATA* (*PI*) (Wan et al., 2020; Yu et al., 2020; Canton et al., 2021; Zhang et al., 2021c). Integrated gene mining techniques have revealed that flowering-related genes are epigenetically regulated in *Prunus* plants. DNA methylation pattern variations were detected in *P. avium* flower buds in early winter (Rothkegel et al., 2020). The chromatin marks of H3K4me3 and H3K27me3 were found in *P. persica* flower buds during endodormancy and ecodormancy, and the expression of *DAM1*, *DAM3*, *DAM4*, and *DAM5* genes were inhibited (Zhu et al., 2020; Canton et al., 2022).

*Prunus* genus consists of over 200 species of flowering trees and shrubs that mostly are deciduous (Kole, 2011; Jung et al., 2018). *P. persica* is one of a few temperate fruit crops that can be grown under diverse climatic conditions (Jung et al., 2018; Wang et al., 2021b). *P. mume*, an early flowering species that blooms in late winter or early spring before new leaves grow (Zhang et al., 2012). Both the species are diploid and highly genetically characterized tree species (Zhang et al., 2012; Verde et al., 2013). Although *P. persica* and *P. mume* are closely related, the flowering time of *P. mume* is much earlier than that of *P. persica* (Sánchez-Pérez et al., 2014). At the same time, there are also differences in flowering time between different varieties of *Prunus* plants (Shi et al., 2020; Yu et al., 2020). In this study, we focused on early flowering in *Prunus* plants. To understand how flowering time is regulated, we integrated comparative genome between *P. persica* and *P. mume* with transcriptional outputs to obtain a potential molecular model for regulating flowering time. We selected transcriptomic data on flowering times of the two *Prunus* species for mutual proof. We propose that flowering time regulation includes at least three processes: cold-adaptation, transcription, and flowering. Our findings not only deepen the understanding of the flowering time control but also extend molecular model of flowering time regulation.

## Materials and methods

### Plant materials

Four *P. persica* genotypes and one *P. mume* cultivar ‘Zao Lve’ were selected in this study. Four *P. persica* genotypes, named A340, A209, A323 and A318, were derived from F_2_ populations constructed by crossing two *P. persica* cultivars (male grandparent ‘Fla.92-2C’ and female grandparent ‘Contender’) showing differences in flowering time. Floral buds from A209 and A340 genotypes were collected at October 14th to 16th, 2015, November 23th, 2015, January 28th, 2016, and February 10th, 2016, respectively. Floral buds from A318 genotype were collected at October 14th to 16th, 2015, November 23th, 2015, January 28th, 2016, February 27th, 2016, and March 12th, 2016, respectively. Floral buds from A323 genotype were collected at October 14th to 16th, 2015, November 23th, 2015, January 28th, 2016, February 27th, 2016, and March 17th, 2016, respectively (Fan et al., 2010; Zhebentyayeva et al., 2013; Yu et al., 2020). *P. mume* cultivar ‘Zao Lve’ had the characteristics of freezing tolerance and early flowering time. Floral buds from ‘Zao Lve’ were collected at November 22th, 2015, December 14th, 2015, January 6th, 2016, and February 18th, 2016, respectively (Zhang et al., 2018).

### Comparative genome analysis

The *P. persica* (v1.0), *P. mume* (wild mei), and *A. thaliana* (TAIR10) genome were obtained from the Genome Database for Rosaceae (GDR), *P. mume* genome project and *Arabidopsis* Information Resource (TAIR), respectively (Zhang et al., 2012; Berardini et al., 2015; Jung et al., 2018). The longest transcripts of the genes were extracted from the genome using Python tools (https://www.python.org/). We performed comparative genome analyses using OrthoFinder software (Emms and Kelly, 2015). The cluster granularity was performed by MCL inflation with default parameters (1.5) (Theodosiou et al., 2008). Then, the sequences were aligned using MAFFT software with FFT-NS-2 method (Katoh and Standley, 2013). Phylogenetic trees of all the orthologous groups were constructed using FastTree software (Price et al., 2009). We further constructed the phylogeny of *P. persica*, *P. mume*, and *A. thaliana* based on single-copy genes. The divergence times of plant species were estimated using the TimeTree website (Kumar et al., 2017). The syntenic relationships of genes in the whole genomes of *P. persica* and *P. mume* were determined based on genome sequences and annotation information. We first performed multiple sequence alignments using BLASTp software (Altschul et al., 1990), and then identified the tandem and collinearity genes using MCScanX software (Wang et al., 2012).

### RNA-seq and gene expression analysis

The read count of the transcriptome was defined as the number of reads compared to the exon in high-throughput sequencing. The read count was obtained using HTseq-count software (Anders et al., 2014). First, we used the GenomicFeatures package included in the R software to convert the count into Fragments Per Kilobase Million (FPKM). The relationships between samples were evaluated using hierarchical clustering analysis (HCA) and principal component analysis (PCA). The identification of differentially expressed genes (DEGs) was carried out according to the method of Audic et al. (Audic and Claverie, 1997). The expression pattern and clustering of DEG were analyzed using the Pheatmap package included in the R software. The shared genes were analyzed and extracted using UpSet included in the EVenn tool and TBtool software, respectively (Chen et al., 2020; Chen et al., 2021). Correlation of gene expression was calculated using the ggplot2 package with the ‘lm’ method included in the R project.

### Gene function annotation and enrichment analysis

We extracted the target sequences using TBtool software (Chen et al., 2020). We searched the target sequences for matching Pfam families using the HMMER website with the parameter set to *E*-value = 1 (Mistry et al., 2021). The relationship between homologous superfamilies and other InterPro entries was calculated by analyzing the overlap between matched sequence sets (Quevillon et al., 2005). The functional annotations and predictions for structure data of the sequences were analyzed using Protein Data Bank in Europe - Knowledge Base (PDBe-KB) (Mir et al., 2018). Gene Ontology (GO) database was used to annotate the cellular component, molecular function, and biological process of sequences (Ashburner et al., 2000). Kyoto Encyclopedia of Genes and Genomes (KEGG) database was used to enrich the pathways of the sequences (Kanehisa and Goto, 2000).

### Transcription factor and gene expression analysis

Genome-wide TFs for *P. persica* were downloaded from the Plant Transcription Factor Database (PlantTFDB) (Jin et al., 2014). We manually retrieved the TFs from the target sequence and further confirmed TFs’ domain signatures using the Pfam database (Mistry et al., 2021). *P. mume* TF orthologues were retrieved based on comparative genome orthologues file. The names, functional descriptions, and GO terms of TFs were analyzed in detail using the UniProt database (The UniProt, 2021). The expression pattern of TF genes was analyzed using the boxplot package included in the R software.

### Molecular model of regulating flowering time analysis

To understand the molecular model of flowering time regulation, we integrated annotation information from multiple databases and the changed into gene expression characteristics. Protein-protein interaction networks and functional enrichment analysis were analyzed according to the orthologs of *A. thaliana* using the STRING database (Pertea et al., 2015). Protein-protein interaction networks were visualized using Cytoscape software (Otasek et al., 2019). Here, we manually filtered KEGG and GO annotation information. The functions of the target sequences were classified based on the functional annotations of the UniProt database (The UniProt, 2021).

## Results

### Genomic characterization of *P. mume* and *P. persica*

**Figure 1:**
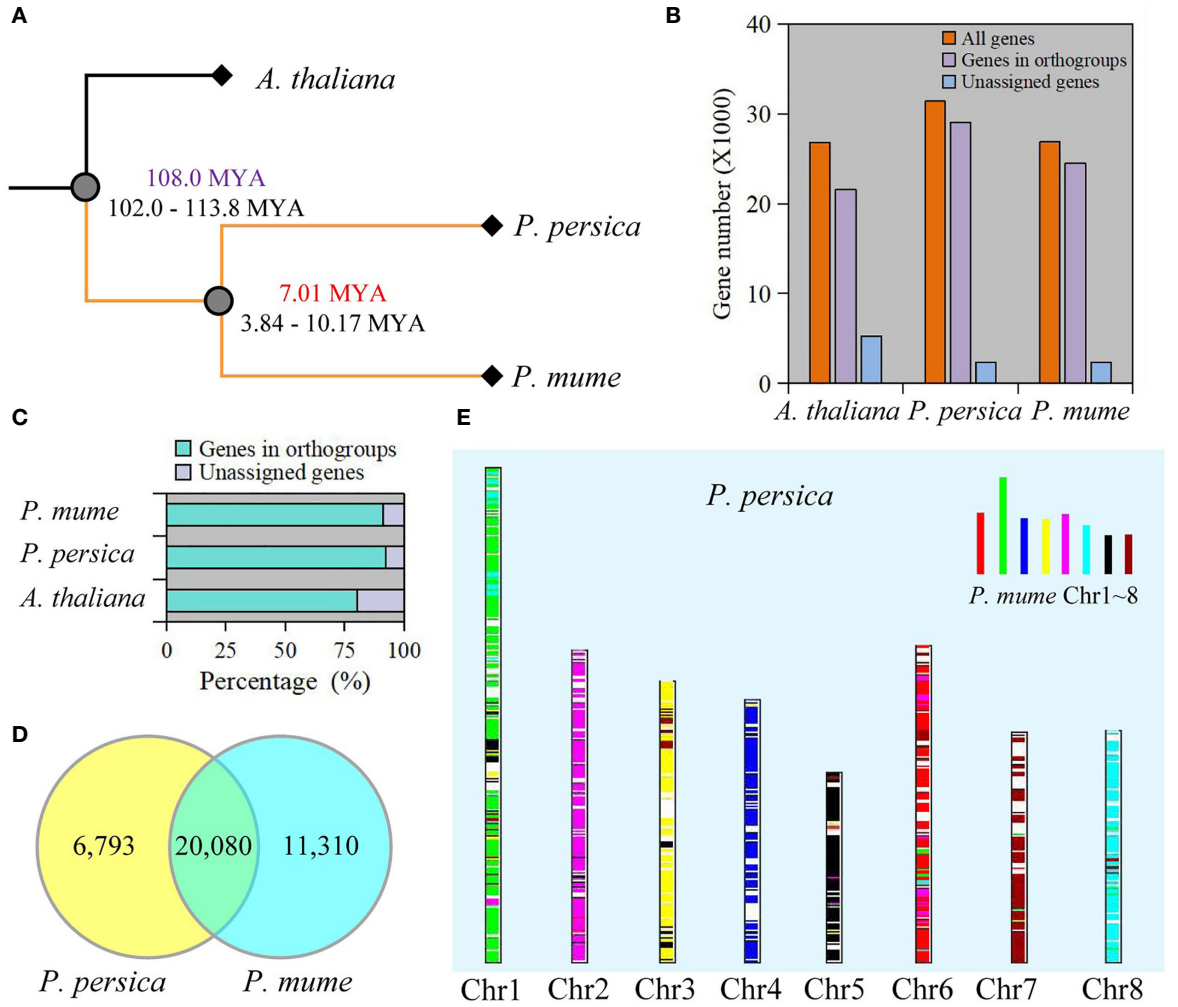
Comparative genomics between *P. mume* and *P. persica.* **(A)** The evolutionary relationships among *A thaliana*, *P. mume*, and *P. persica*. The evolutionary timescale of 102.0-113.8 Mya for the divergence between *A thaliana* and *Prunus*, and 3.84-10.17 Mya for the divergence between *P. mume* and *P. persica*. **(B)** The total number of genes in the genome, the number of genes in orthogroups, and the number of unassigned genes. **(C)** Percentage of genes in orthogroups and unassigned genes. **(D)** Collinearity genes comparison between *P. mume* and *P. persica*. **(E)** Comparison of the *P. persica* genome with the *P. mume.* Syntenic *P. mume* blocks are painted onto *P. persica* chromosomes.

To better understand the genetic background, we compared genome-wide sequences between *P. mume* and *P. persica*, and *A. thaliana* as the outgroup. Using OrthoFinder, 88.2% (75,090 genes) of the total sequences were assigned to 19,299 orthogroups. A total of 10,730 orthogonal groups were identified in *P. mume*, *P. persica*, and *A. thaliana*, of which 6,742 were composed entirely of single-copy genes (**Table S1**). We constructed a species tree by using 1,197 well-supported, non-terminal duplications. The estimated times of divergence for *P. mume* and *P. persica* indicated a relatively recent split (**Figure 1A**). 21,559 genes in *A. thaliana*, 29,020 genes in *P. mume*, and 24,511 genes in *P. persica* were assigned to orthologues (**Figure 1B** and **Table S1**). Moreover, the genes accounting for 92.4% of the total *P. mume* genome and 91.2% of the total *P. persica* genome belonged to orthogroups (**Figure 1C** and **Table S1**). We retrieved 1,952 species-specific orthogroups from all orthogroups, including 1,097 in *A. thaliana*, 569 in *P. mume*, and 286 in *P. persica*. Species-specific orthogroups included 6,120 *A. thaliana* genes, 3,257 *P. mume* genes, and 1,213 *P. persica* genes (**Table S1**). 19,173 orthogroups were found between *P. mume* and *P. persica*, including 20,435 corresponding orthologues (**Table S2**). At the same time, 5,902 resolved gene trees were constructed by orthogroups. 20,080 collinearity genes were identified between *P. mume* and *P. persica* (**Figure 1D**, **Figure S1**, and **Table S3**). 2210 and 1766 tandem duplication of gene pairs were identified in *P. mume* and *P. persica*, respectively (**Tables S4**). A total of 572 syntenic genomic blocks were identified between *P. mume* and *P. persica* (**Figure 1E** and **Figure S2**).

### Transcriptome profiles analysis identified DEGs from dormancy to pre-flowering stage

**Figure 2:**
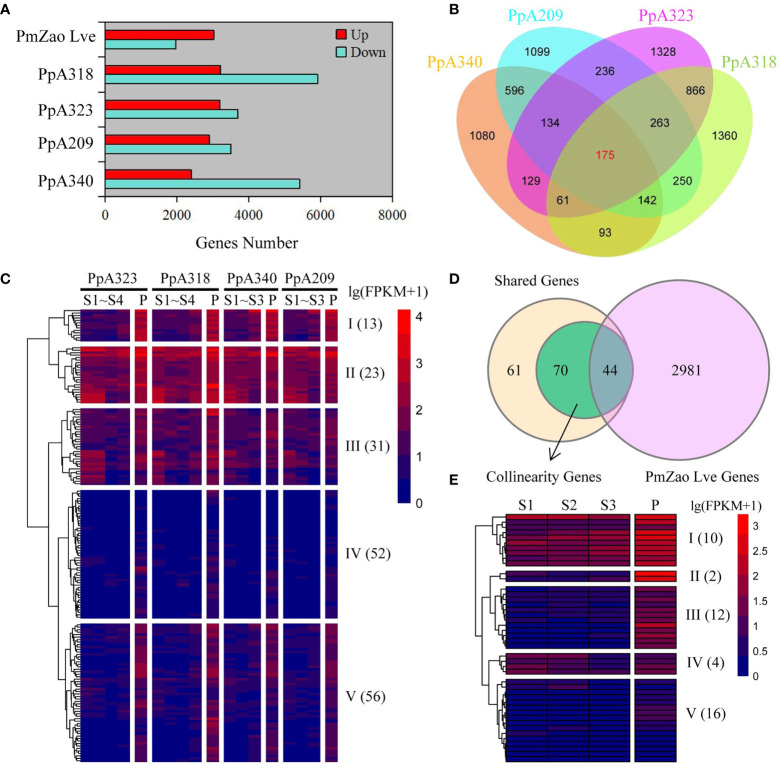
Identification of flowering-related DEGs and gene expression patterns. **(A)** DEGs from dormancy to pre-flowering stage in four *P. persica* genotypes (A209, A340, A318, and A323) and one *P. mume* cultivar (‘Zao Lve’ mei). **(B)** Venn diagram of four *P. persica* genotype DEGs from dormancy to pre-flowering stage. **(C)** Gene expression patterns of shared DEGs in four *P. persica* genotypes. **(D)** Overlap of shared DEGs in *P. persica* and *P. mume* from dormancy to pre-flowering stage with collinearity genes between *P. persica* and *P. mume*. **(D, E)** the gene expression patterns of shared genes in *P. mume*. The letter S stands for dormancy stage and P for pre-flowering stage.

We compared the gene expression levels of *P. mume*, and *P. persica* at the pre-flowering stage with those at the previous stage (dormancy stage). A340, A209, A323, and A318 were derived from F_2_ populations obtained by crossing two *P. persica* cultivars (male grandparent ‘Fla.92-2C’ and female grandparent ‘Contender’) showing differences in flowering time, including A340 and A209. Here, 7,830, 6,401, 6,893, and 9,127 DEGs were identified in the A340, A209, A323, and A318 genotypes, respectively. The number of downregulated DEGs was more than upregulated DEGs in the four *P. persica* genotypes (**Figure 2A**). 1,968 downregulated DEGs and 3,025 upregulated DEGs were identified in the *P. mume* cultivar ‘Zao Lve’ (**Figure 2A**). The upregulated DEGs represented 30.8−46.3% of the total DEGs in the four *P. persica* genotypes from dormancy to pre-flowering. 1,080, 1,099, 1,328, and 1,360 upregulated DEGs were found in the A340, A209, A323, and A318 genotypes, respectively. We found that genotypes with similar flowering time characteristics had more shared genes (**Figure 2B**). 1,047 and 1,365 shared genes were obtained between A340 and A209 and between A323 and A318, respectively. 175 upregulated DEGs were shared in the four *P. persica* genotypes (**Figure 2B**). We extracted the expression data of 175 shared genes into the RNA-seq dataset and standardized the data (**Figure S3** and **Table S5**). The shared DEGs were divided into five clusters based on gene expression patterns (**Table S6**). On the whole, the expression level of 13 genes in cluster I increased gradually from the S1 to P stages. At all stages, the genes in cluster II showed high expression levels, while the genes in cluster IV showed low expression levels. Most of the genes in cluster V were specifically highly expressed in the P stage (**Figure 2C**). Meanwhile, we searched for orthologues of *P. mume* corresponding to 175 shared genes from the four *P. persica* genotypes based on comparative genomic datasets. A total of 185 orthologues were retrieved from *P. mume*, including one-to-one and one-to-many (**Table S7**). 66 orthologues were upregulated from dormancy to pre-flowering stage in *P. mume* (**Figure S4**). Of the 175 shared genes, 114 collinearity genes were obtained between *P. mume* and *P. persica*, and the expression levels of 44 genes were upregulated from dormancy to pre-flowering (**Figure 2D**). These genes had similar expression patterns and were highly expressed at the P stage (**Figure 2E** and **Table S8**). The expression of the three genes (Pm010917, Pm003671, and Pm023232) was not detected in S1 and S2 stages and it gradually increased in the following stages (**Table S8**). Most of the genes in cluster II and III were specifically highly expressed in the P stage. 16 genes belonging to cluster V showed low expression levels (**Figure 2E** and **Table S9**). The shared genes were annotated to a variety of biological processes such as flower development, plant hormone signal transduction and defense response (**Table S10**).

### Mining of early flowering genes from genotypic plants with significant differences in flowering time

**Figure 3:**
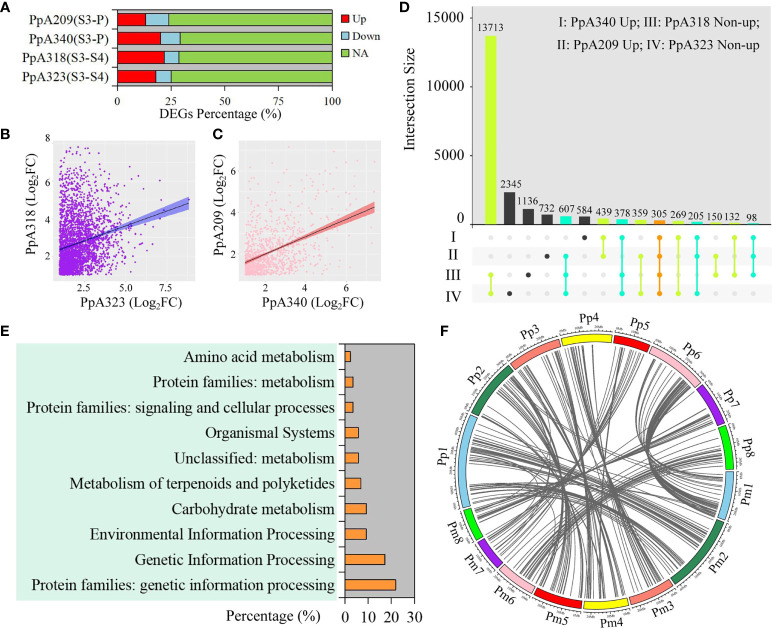
Identification and functional enrichment of early flowering-related DEGs. **(A)** Percentage of DEGs at different stages in four *P. persica* genotypes (A209, A340, A318, and A323). **(B)** Correlation of DEGs between A318 and A323 from S3 to S4 stages. **(C)** Correlation of DEGs between A318 and A323 from S3 to P stages. **(D)** Comparison of genes between pre-flowering stage in the A209 and A340 genotypes and dormancy stage in the A318 and A323 genotypes. Up represents upregulated DEGs, and non-up represents downregulated DEGs and genes with no significant difference change. **(E)** KEGG enrichment analysis of shared genes from **(D)**. **(F)** Collinearity genes of orthologous sequences with shared genes in *P. persica* and *P. mume*.

In order to explore the regulatory genes of early flowering traits, we compared the DEGs with the same chilling requirement in different *P. persica* genotypes, among which A340 and A209 had the flowering ability. The upregulated DEGs accounted for 20.16% and 13.05% of the total genome-wide genes in A340 and A209, respectively (**Figure 3A**). From S3 to S4, although A318 and A323 did not have the flowering ability, the number of upregulated DEGs was higher than that of downregulated DEGs (**Figure 3A**). The fold differential expression of genes showed a positive correlation between genotypes with similar flowering times (**Figures 3B** **, C**). As representative early flowering genotypes, 439 upregulated DEGs were identified in A340 and A209. Here, 18,181 and 16,519 non-upregulated DEGs were extracted from A323 and A318, respectively. We obtained 305 shared genes based on different taxonomic groups of four genotypes (**Figure 3D**). To understand the biological relevance of shared genes, biological pathway enrichment and annotation of these genes were ascertained using KEGG databases. The functional category of shared genes mainly included genetic information processing, environmental information processing, carbohydrate metabolism, and metabolism of terpenoids and polyketides (**Figure 3E**). KEGG pathway enrichment analysis revealed numerous pathways related to environmental information processing, including plant hormone signal transduction, two-component system, MAPK signaling pathway-plant, phosphatidylinositol signaling system, and neuroactive ligand-receptor interaction. We retrieved 87 KO definitions, including methionyl aminopeptidase, plant G-box-binding factor, ABA-responsive element binding factor, MADS-box transcription factor, and protein FLOWERING LOCUS T (**Table S11**). 196 collinearity genes and 283 orthologues of these shared genes were identified in *P. mume* (**Figure 3F** and **Table S12**). Based on the orthologues of these shared genes, we constructed gene expression patterns in *P. mume* (**Figure S5**). We found that these genes had different gene expression patterns, which further narrowed the scope for screening hub genes that regulate early flowering traits.

### Protein-protein interaction networks of early flowering associated proteins

**Figure 4:**
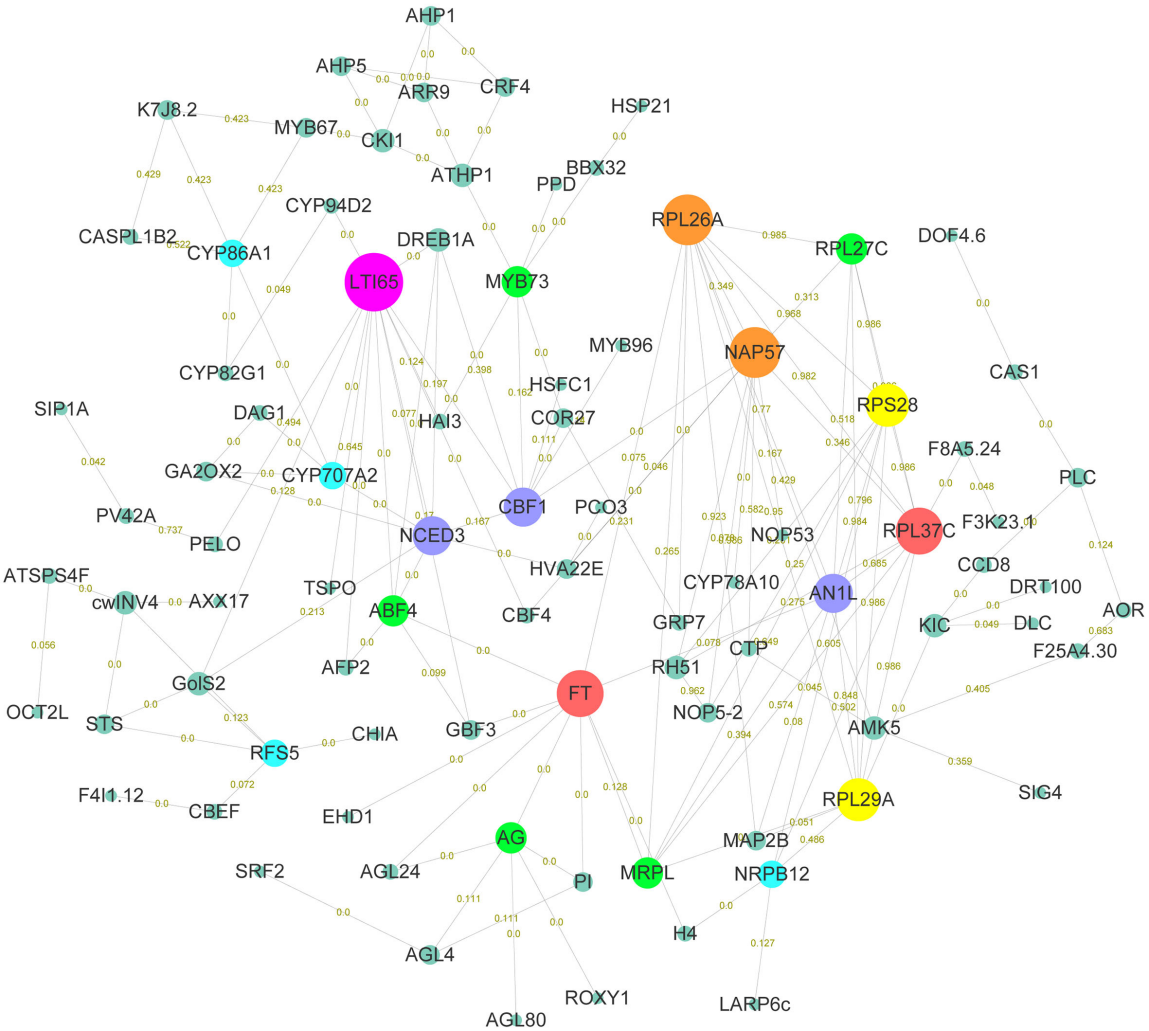
Functional enrichment analysis of early flowering-related genes and protein-protein interaction networks. Proteins are named based on the orthologous sequence of *A. thaliana*. The size of the circle represents the combined score of the STRING database rating. The connectedness is distinguished by different colors. The numbers on the connecting lines represent the coexpression of genes.

In order to explore the gene functions and interactions that potentially regulate early flowering traits, proteins encoded by these candidate genes were annotated and their interactions were predicted. Here, annotation information was obtained for 220 proteins, including transcription factors, kinases, and functional proteins (**Table S13**). The predicted protein-protein interaction network for early flowering traits consisted of 111 proteins, around half of which were transcription factors. In total, 324 protein-protein interactions were found (**Figure 4**). Low-temperature-induced 65 kDa protein (LTI65) was the most interacting protein (13), followed by 60S ribosomal protein L26-1 (RPL26A) and H/ACA ribonucleoprotein complex subunit 4 (NAP57). There were fifteen proteins in the network that interacted with at least six proteins. We found that most proteins were associated with low-temperature stress, protein synthesis, and flowering pathways. Several proteins were involved in the CBF-COR signal transduction pathway, including four members of the dehydration-responsive element-binding protein (DREB) transcription factor family. Ribosomal components represented by members of the RPL family were responsible for the protein synthesis in the cell. FT, as a member of the phosphatidylethanolamine-binding protein (PEBP) family, played a key role in the mobile flower-promoting signal. We identified ten proteins interacting with FT, including floral homeotic protein AGAMOUS (AG), and AGL24. PI, AG, and AGL are the members of the MAD-box family, suggesting that MAD-box and PEBP family members played important roles in regulating early flowering traits. Glutaredoxin-C7 (ROXY1), as a regulator of petal primordia initiation and further petal morphogenesis, forms complex with the AG and regulates flower development. In addition, members of the bZIP transcription factor family (ABF4, GBF4) were also identified in the FT interaction network.

### Transcription factor enrichment

TFs are a class of gene expression regulatory proteins, which play an important role in the regulation of plant flowering. Among the proteins associated with early flowering, TFs were inferred through the PlantTFDB and Pfam databases, respectively. Here, twenty-five TFs covering nine protein families were retrieved, and these TFs were named according to the orthologues following the rules for *A. thaliana* (**Table 1**). We identified six MADS-box family members, including AGL4, AGL24, AGL80, AG, PI, and SVP. MADS-box TFs interact with several transcriptional targets to mediate a diverse range of plant processes, including differentiation, flowering, transcription, and transcription regulation (**Table 1** and **Table S14**). Twenty-four percent of all the identified TFs were apetala2/ethylene response factor (AP2/ERF) homologs, including ERF, RAV, and CBF/DREB subfamilies. Other TFs, including MYB, WRKY, bZIP, heat shock factor (HSF), homeobox protein (ATH), dof zinc finger protein (DOF), and auxin response factor (ARF), comprised 12%, 4%, 8%, 8%, 4%, 8%, and 4% of the total candidate TFs, respectively. These TFs regulate hormone signal transduction and synthesis pathways, including auxin, abscisic acid, ethylene, cytokinin-activated signaling pathway, and gibberellin biosynthetic process (**Table 1** and **Table S14**). DAG1 and FAR1 were involved in response to red or far-red light. FAR1 negatively regulates leaf senescence and positively regulates circadian rhythm (**Table S14**). Among TFs involved in abiotic stress response, WRKY, MYB, and AP2/ERF represented a large protein family, respectively, which displayed diverse roles in various biological processes (**Table S14**). ATH1, as a specific activator of *FLC* expression, controls floral competency. ATH1 was involved in several biological processes, such as floral organ abscission, photomorphogenesis, regulation of gibberellin biosynthetic process, and vegetative to the reproductive phase transition of the meristem (**Table S14**).

**Table 1:** Identification and functional annotation of early flowering-related TFs.

*P. persica*	Orthologues in *P. mume*	Family	Preferred Name	Biological process
Prupe.2G213000	Pm018533	ARF	ARF16	Transcription regulation; Auxin signaling pathway
Prupe.2G182800	Pm018422	bZIP	GBF3	Transcription regulation
Prupe.8G126600	Pm021430	bZIP	ABF4	Transcription regulation; Abscisic acid signaling pathway
Prupe.2G192300	Pm018337	Dof	DAG1	Transcription regulation; Response to red or far red light
Prupe.2G314800	Pm019710	Dof	DOF4.6	Transcription regulation
Prupe.5G089900	Pm023777	AP2/ERF	CBF4	Transcription regulation; Abscisic acid signaling pathway; Stress response
Prupe.5G090600	Pm023768	AP2/ERF	ERF025	Transcription regulation; Ethylene signaling pathway
Prupe.5G117800	Pm024053	AP2/ERF	ERF061	Transcription regulation; Ethylene signaling pathway
Prupe.5G090000	Pm023773	AP2/ERF	CBF1	Transcription regulation; Stress response
Prupe.5G090100	Pm023775	AP2/ERF	DREB1A	Transcription regulation; Stress response
Prupe.3G019900	Pm013049	AP2/ERF	CRF4	Transcription regulation; Cytokinin signaling pathway; Ethylene signaling pathway
Prupe.1G074100	Pm004420	FAR1	FAR1	Transcription regulation; Red or far-red light signaling pathway; Circadian rhythm; Leaf senescence
Prupe.7G206900	Pm027197	HSF	HSFA6B	Transcription regulation; Stress response
Prupe.7G231100	Pm027421	HSF	HSFC1	Transcription regulation; Stress response
Prupe.1G531600	Pm004416	MADS-box	SVP	Transcription regulation; Differentiation; Flowering
Prupe.1G290500	Pm030595	MADS-box	AGL4	Transcription regulation; Differentiation; Flowering
Prupe.1G489400	Pm004718	MADS-box	PI	Transcription regulation; Differentiation; Flowering
Prupe.2G109500	Pm017464	MADS-box	AGL80	Transcription regulation
Prupe.3G111300	Pm014563	MADS-box	AG	Transcription regulation; Differentiation; Flowering
Prupe.1G531500	Pm004417	MADS-box	AGL24	Transcription regulation; Differentiation; Flowering
Prupe.4G126900	Pm010927	MYB	MYB67	–
Prupe.3G268000	Pm015880	MYB	MYB96	Transcription regulation; Abscisic acid signaling pathway; Plant defense; Stress response
Prupe.1G441700	Pm005220	MYB	MYB73	Transcription regulation
Prupe.1G413700	Pm005487	TALE	ATH1	Transcription regulation
Prupe.3G270800	Pm015851	WRKY	WRKY72	Transcription regulation

**Figure 5:**
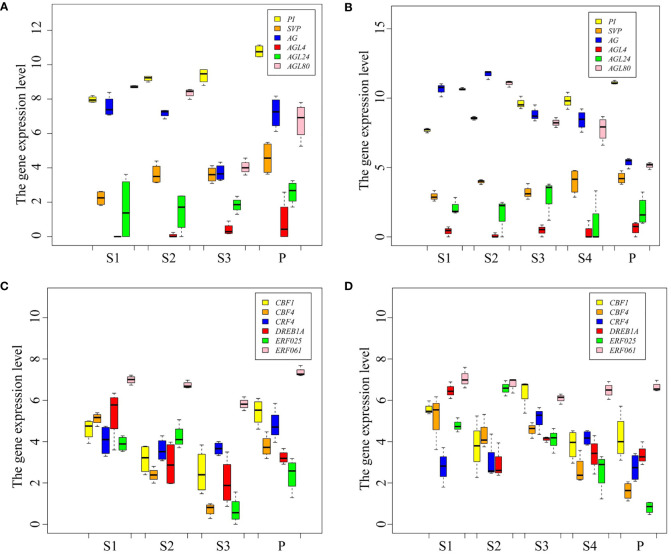
Expression patterns of transcription factor genes. **(A, B)** Expression levels of MAD-box TF genes in the early and late flowering genotypes, respectively. **(C, D)** Expression levels of AP2/ERF TF genes in the early and late flowering genotypes, respectively.

MADS-box family members are the key TFs for flowering, and AP2/ERF family members play an important role in plant response to cold stress. Here, the expression patterns of MADS-box and AP2/ERF TF coding genes were analyzed in *P. persica* (**Figure 5**). The expression level of *PI* and *AGL4* genes showed an upward trend from S1 to P stages in the early and late flowering genotypes. *SVP* gene showed different expression patterns in the early and late flowering genotypes. The expression of *SVP* was the highest in A340 genotype at the P stage, while at S4 stage the highest expression was observed in A318 genotype. The expressions of *AG* gene showed a changing trend from dropping at first to rising afterwards in early flowering genotypes. However, the expression of *AG* gene in late flowering genotypes showed a continuous downward trend. The expression of *AGL80* gene showed the opposite trend in the two genotypes of *P. persica*. The expression of *AGL80* gene in the P stage was the highest, which was 6.9 and 2.3 times higher than the expression in S1 and S3 stages, respectively (**Figures 5A, B**). *ERF061* gene had a high basic expression level in the early and late flowering genotypes. The expressions of *ERF061*, *CBF4*, *CBF1*, *CRF4*, and *DREB1A* gene showed a changing trend from dropping at first to rising afterwards in early flowering genotypes. The expression pattern of these genes in late flowering was not as regular as that in early flowering genotypes. Overall, AP2/ERF TF coding genes were upregulated from S3 to P stages in early flowering genotypes. In late flowering genotypes, the expression of these genes showed a downward or constant trend from S3 to S4 stages and continued to the P stage (**Figures 5A, B**).

### Potential molecular model of flowering time regulation

**Figure 6:**
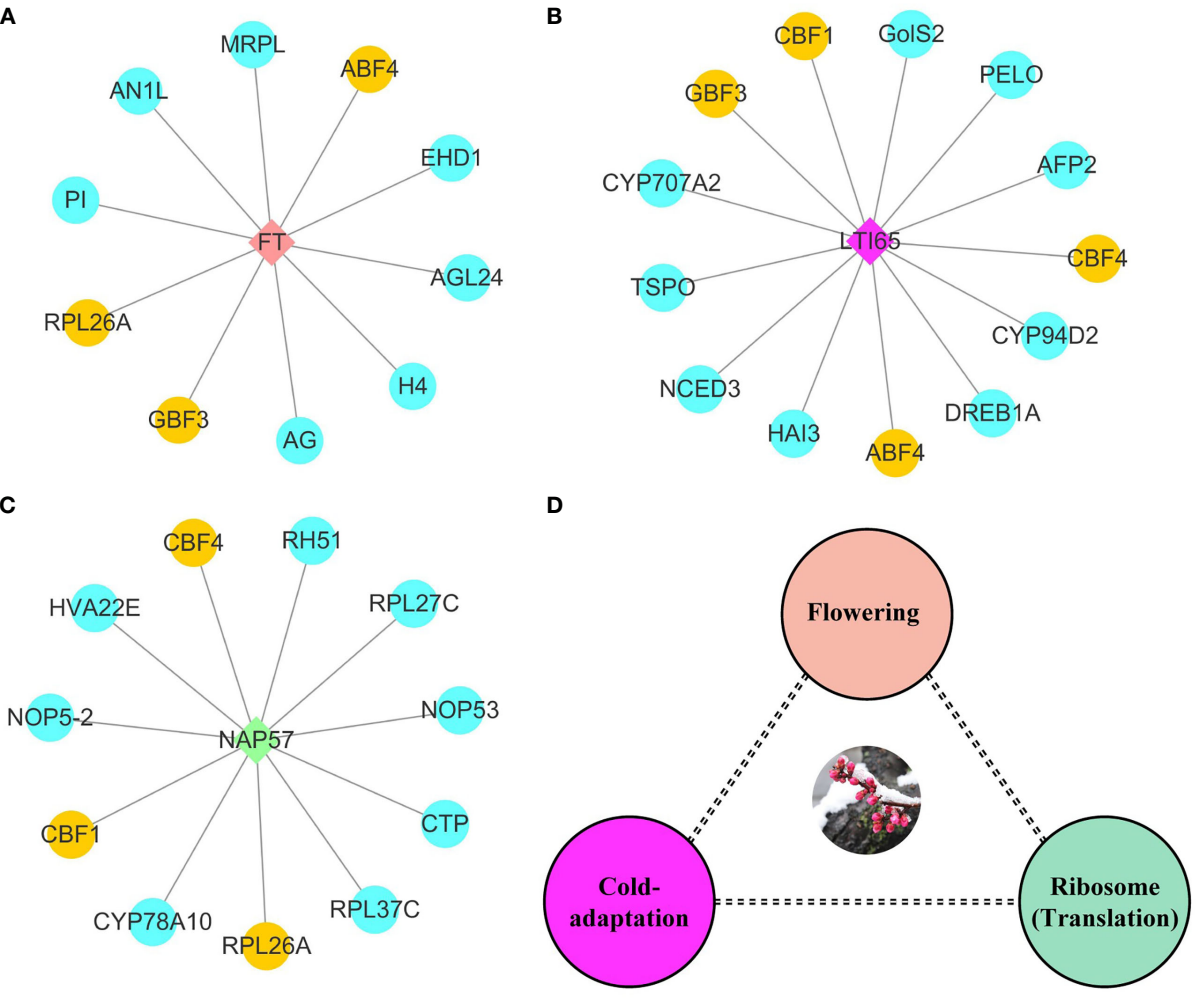
Potential hub regulatory protein interactions and models for early flowering traits. **(A)** Interaction network with FT as hub protein in early flowering associated proteins. **(B)** Interaction network with LTI65 as hub protein in early flowering associated proteins. **(C)** Interaction network with NAP57 as hub protein in early flowering associated proteins. The yellow represents shared proteins in the three interaction networks. The blue represents the unique proteins in each interaction network. **(D)** Potential models for regulating flowering time.

To investigate the molecular model of flowering time regulation, we selected three hub protein sets *via* connectivity in protein-protein interaction networks. FT belonged to the phosphatidylethanolamine-binding protein family and was involved in biological processes such as flower development, meristem determinacy, photoperiodism, flowering, and cell differentiation. FT had protein-protein interaction with ten proteins, including three MADS-box TFs (AG, AGL24, and PI) and two bZIP TFs (ABF4 and GBF3) (**Figure 6A**). GBF3 TF also had protein-protein interaction with LTI65 protein. Most proteins in LTI65 protein-protein interaction were closely related to abiotic stress. We identified three AP2/ERF TFs (CBF1, CBF4, and DREB1A), in addition to ninja-family protein (AFP2), 9-cis-epoxycarotenoid dioxygenase (NCED3), translocator protein homolog (TSPO) and Galactinol synthase 2 (GolS-2) proteins (**Figure 6B**). Nopp-140-associated protein of 57 kDa homolog (NAP57) catalyze pseudouridylation of rRNA, which plays a central role in ribosomal RNA processing. The NAP57 protein-protein interaction network shared CBF1, CBF4, and 60S ribosomal protein L26-A (RPL26A) with the FT and LTI65 protein-protein interaction networks. RPL26A, RPL27C, and RPL37C were annotated as the ribosomal proteins for molecular function and were involved in the biological process of transcription. Ribosome biogenesis proteins (NOP53 and NOP5-2) were associated with numerous RNAs including the 27S and 7S pre-rRNAs and the box H/ACA snoRNA snR37 (**Figure 6C**). FT, LTI65, and NAP57 protein-protein interaction networks were mainly involved in the molecular regulation of flowering, cold response and RNA transcription, respectively. Here, we constructed a potential molecular model for flowering time regulation. The molecular model of flowering time regulation included at least three processes: cold-adaptation, transcription, and flowering (**Figure 6D**).

## Discussion

Flowering time is regarded as an environmental adaptive trait of plants, which is crucial for the survival and reproduction (Ding et al., 2020; Gaudinier and Blackman, 2020). The flowering regulation of *Prunus* is closely related to the heat and chilling requirements, and bud dormancy, including endodormancy and ecodormancy (Sánchez-Pérez et al., 2012; Castède et al., 2014; Zhang et al., 2018; Calle et al., 2019; Yu et al., 2020). The dormancy release is the starting point of flowering process (Bielenberg et al., 2008; Zhang et al., 2018). The dormancy process is also accompanied by the chilling requirement in winter (Lang et al., 1987; Campoy et al., 2012). The plant can bloom under suitable environmental conditions when chilling requirements are met (Campoy et al., 2012; Castède et al., 2014). The flowering time varies by species and cultivars (Dirlewanger et al., 2012). *Prunus mume*, an early flowering plant, blooms in late winter or early spring before new leaves grow (Zhang et al., 2012). The *P. persica* genotypes are derived from F_2_ populations constructed by crossing two *P. persica* cultivars (male grandparent ‘Fla.92-2C’ and female grandparent ‘Contender’) showing differences in flowering time (Yu et al., 2020).

With all the important advances in genomic sequencing, it is extremely important to understand the biological significance of a variety of sequence and structure data in the post-genome era. So far, the genomes of seventeen *Prunus* species have been published according to the plaBiPD database (https://www.plabipd.de/). The *P. persica* and *P. mume* genomes have been sequenced and assembled in five and three versions, respectively (Zhang et al., 2012; Verde et al., 2013; Guan et al., 2021; Wang et al., 2021b; Zhang et al., 2021a; Zheng et al., 2021; Lian et al., 2022). 10,704 shared orthologues have been obtained across the six Rosaceae species (*Malus×domestica*, *Rosa chinensis*, *Prunus yedoensis*, *P. persica*, *P. armeniaca* and *P. mume*) (Zheng et al., 2021). Species evolution suggests that *P. persica* and *P. mume* are closely related and lacks recent whole genome duplication (WGD) events (Verde et al., 2017; Jiang et al., 2019; Zheng et al., 2021). In this study, we identified 19,173 orthogroups and 20,080 collinearity genes between *P. mume* and *P. persica*. 20,435 and 25,185 orthologues were retrieved in the *P. mume* and *P. persica* genomes, accounting for 76.04% and 80.23% of the total genome, respectively. In addition, the Pan-genome of *Prunus* was assembled based on the 377 accessions of *Prunus* germplasm in the Himalayas (Wang et al., 2021b). The molecular mechanism of endodormancy to ecodormancy transition is studied by combining *P. persica* and *P. armeniaca* (Yu et al., 2020). These results provide further evidence that *P. mume* and *P. persica* are closely related.

In the past 20 years, the research and application of transcriptome technology have gained tremendous importance in the mining of key genes and transcriptional regulation mechanism related to specific traits. The combined genomic and transcriptome strategy provides insight into the molecular mechanism underlying important plant-specific traits, such as the carotenoid metabolism of *Lonicera japonica*, tanshinones synthesis of *Salvia miltiorrhiza*, and tortuous-branch phenotype of *P. mume* (Pu et al., 2020; Ma et al., 2021; Zheng et al., 2021). 59 candidate genes related to flowering time and flower development were screened from the flower development processes by comparing the transcriptomes of the two *Solanum lycopersicum* genotypes (Wang et al., 2021a). 4,871 and 5,319 DEGs were identified during endodormancy release in *P. armeniaca* low and high chilling requirement genotypes, respectively (Canton et al., 2021). Meanwhile, 5,912 and 7,048 DEGs were identified during endodormancy release in *P. persica* low and high chilling requirement genotypes, respectively (Canton et al., 2021). We identified 7,830, 6,401, 6,893, and 9,127 DEGs in the four *P. persica* genotypes from dormancy to pre-flowering. 1,968 downregulated DEGs and 3,025 upregulated DEGs were identified in the *P. mume* cultivar ‘Zao Lve’. DEGs represented 20.6% to 33.0% of the total number of genes in the genome from dormancy to pre-flowering. A large number of genes were differentially expressed in the flowering process of *Prunus* plants, suggesting that the flowering is regulated by a complex mechanism regulated by multiple genes.

Flowering pathways are constantly being developed in plants. The integrators of flowering time play a crucial role in this process (Lloret et al., 2018). At present, FT, LFY, FLC, and SOC1 are thought to be important integrators of floral meristem genes, such as the *CAULIFLOWER* (*CAL*), *FRUITFUL* (*FUL*), and *AP1* (Simpson and Dean, 2002; Posé et al., 2012; Khan et al., 2014; Ó'Maoiléidigh et al., 2014). In *Prunus* plant, MADS-box TF family members are significantly associated with flowering time and dormancy (Bielenberg et al., 2008; Kai et al., 2018; Zhang et al., 2018; Quesada-Traver et al., 2020). SVP, PI, AG, and AGL24 TFs, belonging to the MADS-box family, directly or indirectly regulate *FT* gene. Meanwhile, many plant hormone related genes have been identified in the flowering pathway, such as the pyrabactin resistance (PYR) and 9-cis epoxycarotenoid dioxygenase (NCED) genes of the abscisic acid pathway, GA 20-oxidase (GA20ox) and GA 3-oxidase (GA3ox) genes of the gibberellin pathway, and 3-epi-6-deoxocathasterone 23-monooxygenase (CYP) gene of brassinosteroid pathway (Canton et al., 2021; Zhang et al., 2021c). In this study, we identified six MADS-box family members, including AGL4, AGL24, AGL80, AG, PI, and SVP from dormancy to pre-flowering. MADS-box TFs interact with several transcriptional targets to mediate a diverse range of plant processes. Twenty-four percent of all the identified TFs were AP2/ERF homologs, including ERF, RAV, and CBF/DREB subfamilies. Other TFs included MYB, WRKY, bZIP, HSF, ATH, DOF, and ARF TFs. These TF genes have been shown to involve flowering regulation, especially AP2/ERF TFs (Reinoso et al., 2002; Julian et al., 2011; Goeckeritz and Hollender, 2021).

Usually, *Prunus* plants flower during transition from endodormancy to ecodormancy (Castède et al., 2014; Gaudinier and Blackman, 2020; Canton et al., 2022). Flower buds complete their chilling requirements during endodormancy (Fan et al., 2010; Castède et al., 2014). Although low temperature inhibited the expression of *SVP* and *FLC* genes, which were negative regulators of *FT*, it also inhibited other genes that promoted flowering. The ability of plants to adapt to cold environment was one of the key factors for flowering. The effect of low temperature on plant flowering plays different roles in different stages. Toward the end of winter, low temperature is the main cause of growth arrest during ecodormancy (Lang et al., 1987; Luedeling, 2012; Khan et al., 2014). Moreover, temperature affects the transcriptional ability of genes, which is essential for the morphological transformation of plant tissues during flowering. In this study, the hub proteins, including FT, LTI65 and NAP57 were enriched in CBF-COR pathway, transcription, and flowering. We, therefore, propose a molecular model that covers three aspects of flowering time regulation: cold-adaptation, transcription, and flowering. These results suggest that the regulation of flowering time in plants is influenced by both hereditary substances and environmental conditions.

## Data availability statement

The original contributions presented in the study are publicly available. This data can be found here: NCBI BioProject PRJNA714446 and NGDC (https://bigd.big.ac.cn/) BioProject PRJCA000291.

## Author contributions

PL and XZ conceived and designed the experiments. JW and HY provided the data collection and analysis platform. PL analyzed the data and wrote the manuscript. LL and BS provided help with the experiments. QZ, LL, BS, HY, JW, and XZ examined and finalized the manuscript. All authors read and approved the final manuscript.

## Funding

This research was funded by the Natural Science Foundation of Hebei Province under grant No. C2021204113 and C2021204184.

## Conflict of interest

The authors declare that the research was conducted in the absence of any commercial or financial relationships that could be construed as a potential conflict of interest.

## Publisher’s note

All claims expressed in this article are solely those of the authors and do not necessarily represent those of their affiliated organizations, or those of the publisher, the editors and the reviewers. Any product that may be evaluated in this article, or claim that may be made by its manufacturer, is not guaranteed or endorsed by the publisher.
