# Supplementary material for: Integration of genome and transcriptome reveal molecular regulation mechanism of early flowering trait in Prunus genus (Prunus mume and Prunus persica)
Source: Front Plant Sci. 2022 Oct 6;13:1036221. doi: 10.3389/fpls.2022.1036221 (PMC9582937; doi:10.3389/fpls.2022.1036221)
Supplement: Supplementary file 1 [file DataSheet_1.zip › Supplementary Figures.docx]

Supplementary Material

# 1 Supplementary Figures and Tables

## 1.1 Supplementary Figures


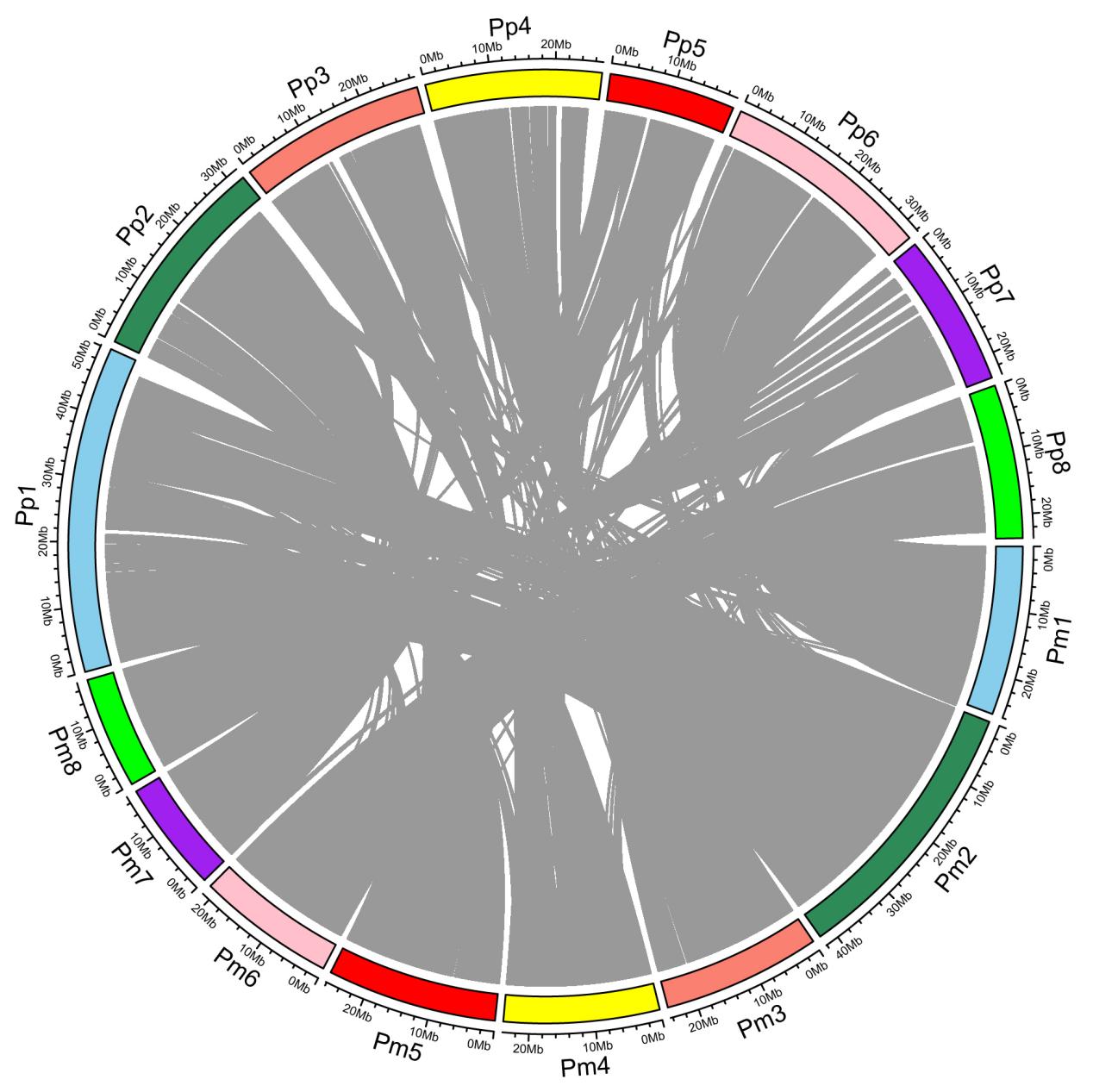


Figure S1. Synteny patterns between genomic regions from *P. persica* and *P. mume*.


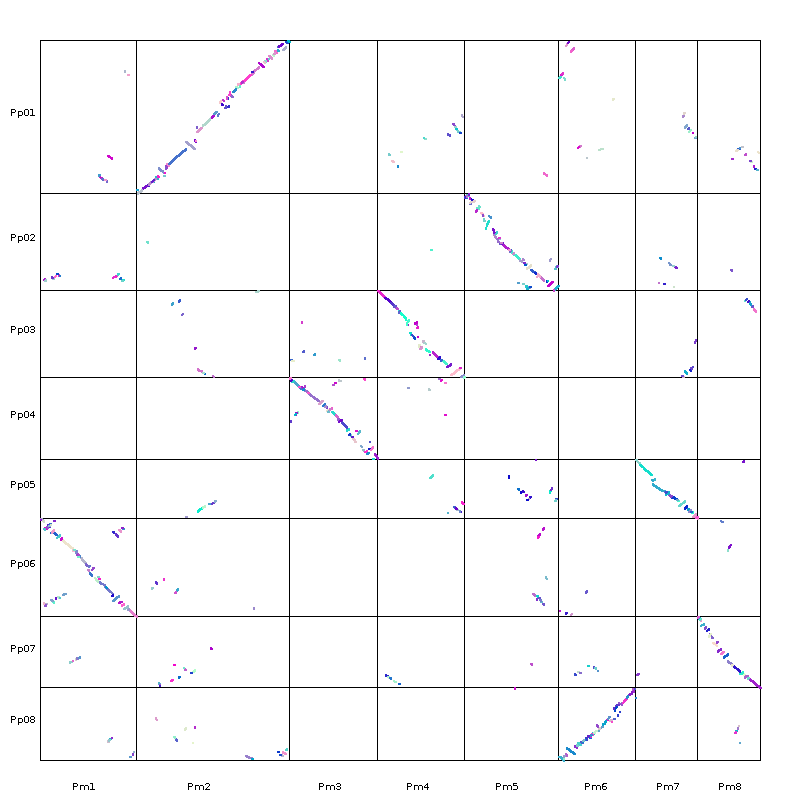


Figure S2. Syntenic blocks between between genomic regions from *P. persica* and *P. mume*.


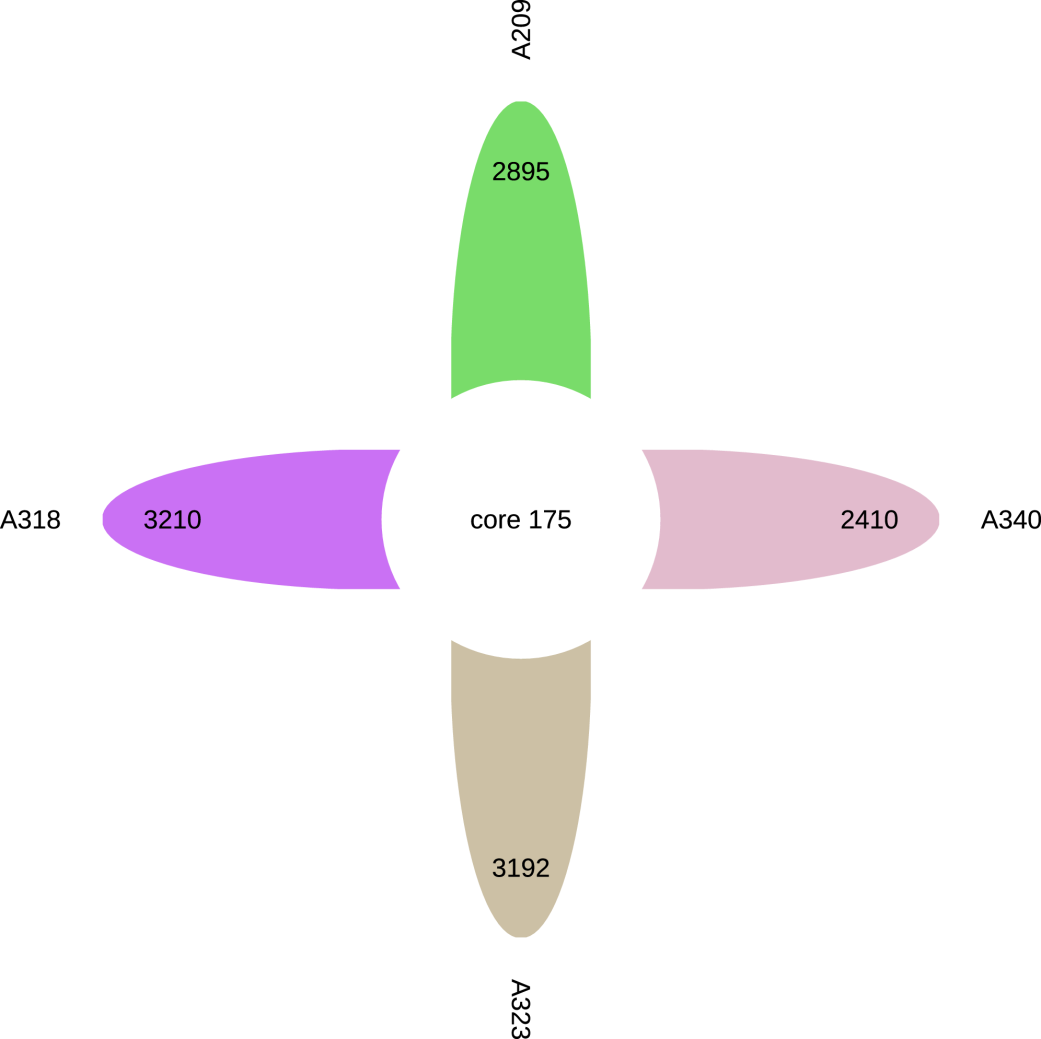


Figure S3. Comparative analysis of up-regulated genes from dormancy to pre-flowering stage in four *P. persica* genotypes (A340, A209, A323, and A318).


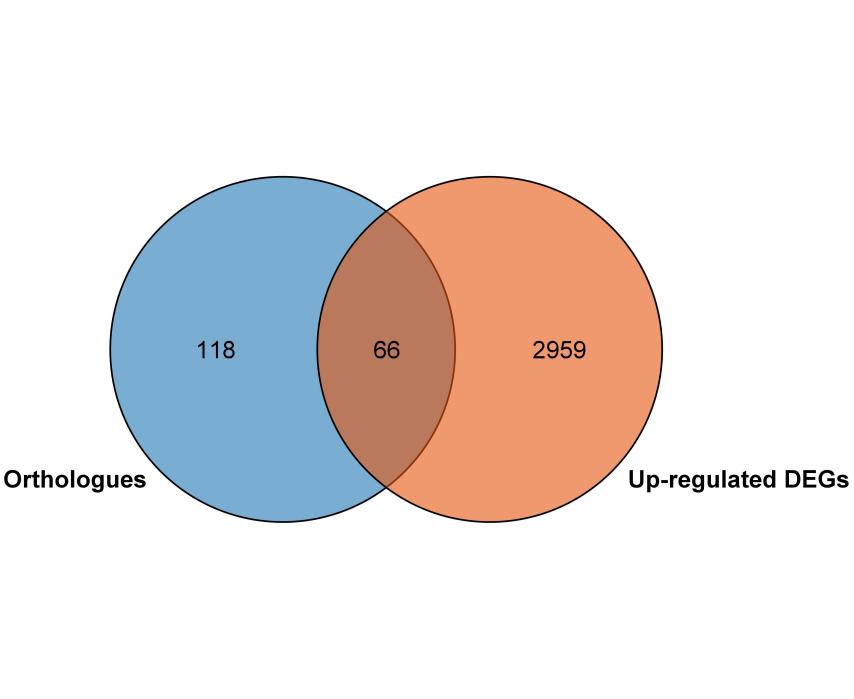


Figure S4. Overlap of orthologues of shared genes and up-regulated DEGs in *P. mume* from dormancy to pre-flowering stage.


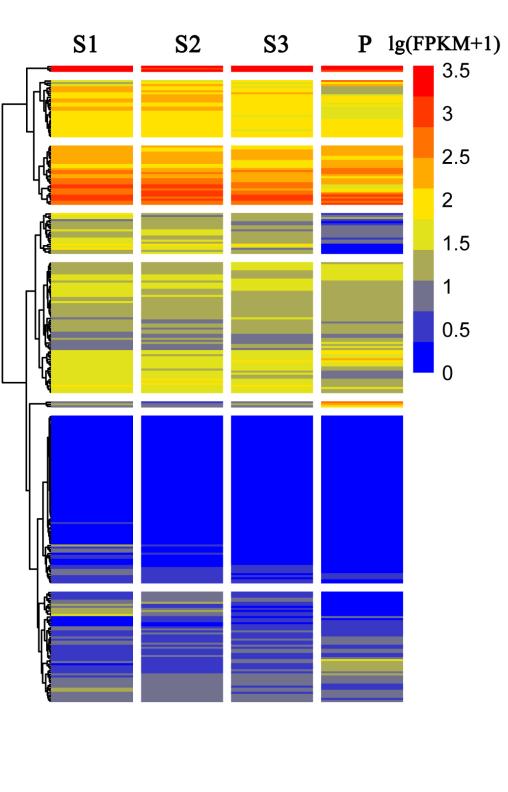


Figure S5. The gene expression patterns of orthologues with *P. persica* shared genes in *P. mume*.

## 1.2 Supplementary Tables

Table S1. Comparative genomics statistics in *A. thaliana*, *P. mume* and *P. persica*.

Table S2. Orthologues between *P. persica* and *P. mume*.

Table S3. Chromosomal distribution and potentially collinearity duplication in *P. mume* and *P. persica*.

Table S4. Tandem duplication of gene pairs in *P. mume* and *P. persica*.

Table S5. Expression profiles of 175 shared genes in the four *P. persica* genotypes (A340, A209, A323, and A318).

Table S6. The clusters of shared DEGs based on gene expression patterns in *P. persica*.

Table S7. 175 shared genes of the four *P. persica* genotypes correspond to orthologues of *P. mume*.

Table S8. Expression profiles of shared genes in the *P. mume* cultivar 'Zao Lve'.

Table S9. The clusters of shared DEGs based on gene expression patterns in *P. mume*.

Table S10. Annotation of shared genes based on the STRING databases.

Table S11. KO annotation of shared genes based on the KEGG databases.

Table S12. Collinearity genes and orthologues of shared genes in *P. mume*.

Table S13. Functional annotation of shared genes based on the STRING databases.

Table S14. GO annotations of the transcription factor.
